# Prognostic Factors in Amyotrophic Lateral Sclerosis: A Population-Based Study

**DOI:** 10.1371/journal.pone.0141500

**Published:** 2015-10-30

**Authors:** Mirian Conceicao Moura, Maria Rita Carvalho Garbi Novaes, Emanoel Junio Eduardo, Yuri S. S. P. Zago, Ricardo Del Negro Barroso Freitas, Luiz Augusto Casulari

**Affiliations:** 1 Hospital Regional da Asa Norte, State Secretariat of Health of the Federal District, Brasilia, DF, Brazil; 2 School of Health Sciences, DF, and University of Brasilia, Brasilia, Brazil; 3 School of Health Sciences, Brasilia, DF, Brazil; 4 University Hospital of Brasília, Brasilia, DF, Brazil; Macquarie University, AUSTRALIA

## Abstract

**Objective:**

To determine the prognostic factors associated with survival in amyotrophic lateral sclerosis at diagnosis.

**Methods:**

This retrospective population-based study evaluated 218 patients treated with riluzole between 2005 and 2014 and described their clinical and demographic profiles after the analysis of clinical data and records from the mortality information system in the Federal District, Brazil. Cox multivariate regression analysis was conducted for the parameters found.

**Results:**

The study sample consisted of 132 men and 86 women with a mean age at disease onset of 57.2±12.3 years; 77.6% of them were Caucasian. The mean periods between disease onset and diagnosis were 22.7 months among men and 23.5 months among women, and the mean survival periods were 45.7±47.0 months among men and 39.3±29.8 months among women. In addition, 80.3% patients presented non-bulbar-onset amyotrophic lateral sclerosis, and 19.7% presented bulbar-onset. Cox regression analysis indicated worse prognosis for body mass index (BMI) <25 kg/m^2^ (relative risk [RR]: 3.56, 95% confidence interval [CI]: 1.44–8.86), age >75 years (RR: 12.47, 95% CI: 3.51–44.26), and bulbar-onset (RR: 4.56, 95% CI: 2.06–10.12). Electromyography did not confirm the diagnosis in 55.6% of the suspected cases and in 27.9% of the bulbar-onset cases.

**Conclusions:**

The factors associated with lower survival in amyotrophic lateral sclerosis were age >75 years, BMI <25 kg/m^2^, and bulbar-onset.

## Introduction

Amyotrophic lateral sclerosis (ALS) is a degenerative disease characterized by the progressive loss of upper and lower motor neurons in the brain and spinal cord and progresses with muscle weakness and atrophy with or without pyramidal syndrome. Its incidence is estimated at 2.08 per 100,000 person-years, and the average age at symptom onset is 61.8±3.8 years (range of 54–67 years)[1].

The disease is associated with poor prognosis, and its etiology has been attributed to the temporal interaction between genetic and environmental factors [2,3]. Death is due to respiratory failure, with an average survival period after diagnosis of 24–36 months [3].

With respect to the clinical trials of treatments aimed at increasing survival, only riluzole, an anti-glutamatergic drug, exhibited moderate efficacy[4]. The possible causes of the failure of the clinical trials include clinical heterogeneity of the disease, because ALS may represent a spectrum of diseases with diverse causes and etiologies, and there is also difficulty in identifying evolution and prognostic factors [5].

Moreover, considering that no effective therapy is available for ALS, the establishment of a prognosis during diagnosis is important for the development of a treatment plan for each patient.

The present study aimed to determine the factors associated with survival in ALS at the time of diagnosis.

## Methods

This descriptive, population-based study was conducted using retrospective data from the medical records of patients treated at the Reference Center for Neuromuscular Diseases (CRDN) located in the Federal District, Brazil, and from riluzole-dispensing records between 2005 and 2014.

All patients selected for the study were treated with riluzole. According to Ministry Of Health of Brazil protocol[6], only patients with definite or probable diagnosis in accordance with the El Escorial diagnostic criteria [7, 8] are allowed to use riluzole. They were enrolled in the study after excluding the diagnosis of other diseases.

The data extracted included age, sex, time of onset of symptoms, onset site, form of progression to another affected limb, family history of ALS, presence of pyramidal syndrome, time between onset of symptoms and diagnosis, disease course duration, self-reported weight and height, diagnosis confirmed by electromyography, smoking, alcohol consumption, contact with pesticides or heavy metals, intense physical activity (athlete), history of malignancy, associated dementia, and a set of socioeconomic indicators included in the Municipal Human Development Index in 2010 (IDHM-2010)[9].

Disease onset was regarded as the time from symptom onset to the endpoint or disease course duration in months, or the time from symptom onset to death or indication for tracheostomy.

Dementia was defined as the story of cognitive and behavioral deficits according to Lund and Manchester Groups criteria [10] in patient records and frontotemporal atrophy in neuroimaging.

The database from the Mortality Information System of the Unified Health System (SIM/DATASUS) was reviewed for the same period, and the data were cross-referenced with those collected in the CRDN to confirm the dates of death for the patients.

The BMI at the onset of symptoms was calculated as weight (kg)/height^2^ (m^2^).[11]

### Statistical analysis

Descriptive statistics were performed using two-tailed Student’s t-tests and χ2 tests at a significance level of 5%. Multivariate analysis was performed, including all variables. Initially, Cox univariate regression analysis was used for socio-demographic and clinical variables in relation to the survival period. Variables with p<0.25 in the univariate analysis [12] were included in the Cox multivariate regression analysis. The final multivariate regression model was built by the successive exclusion of each variable from the initial multivariate model. The likelihood ratio test was used to evaluate the importance of each excluded variable [12]. The level of significance was set at 5%. The survival functions for the patients were estimated using the Kaplan-Meier method and were compared using the log-rank test. All analyses were performed using Statistical Analysis System software version 9.3 and Statistical Package for the Social Sciences software version 19.0.

The study was approved by the Research Ethics Committee of Foundation of Teaching and Research in Health Sciences -FEPECS in the Federal District, Brazil, under protocol No. 820.117/2014. The Ethics Committee waived the written consent form because the study consisted of analysis of patient records (many of patients were deceased) and secondary databases (mortality data).

## Results

From 2005 to 2014, 145 medical records and 73 riluzole-dispensing records were found, totaling 218 cases. The adjusted incidence rate for the population aged >20 years in the Federal District according to the IBGE census of 2010[13] was 1.26 cases per 100,000 person-years, considering a population of 1,740,922 inhabitants. The adjusted incidence rate for the population aged >45 years was 2.38 cases per 100,000 person-years, considering a population of 925,334 inhabitants.

**Fig 1:**
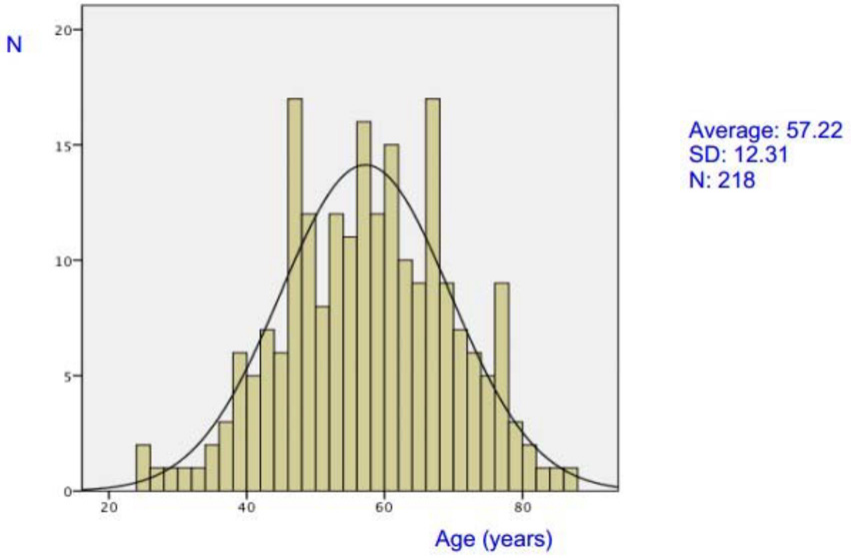
Age distribution in patients with amyotrophic lateral sclerosis.

The study sample consisted of 132 men (60.6%) and 86 women (39.4%), but this sex difference was not significant (p = 0.06). The frequency distribution according to sex is shown in Fig 1. The mean age considering both sexes was 57.2±12.3 years, with a range of 25–86 years. The average ages were 56.3±12.3 years among men and 58.6±12.3 years among women, without a significant difference between the sexes (p = 0.179).

A retrospective analysis of the records showed that 109 patients (50%) were classified as Definite; 41 (18.8%) were Probable; 50 (22.9%) were Possible and 18 (8.3%) were Suspect.

With regard to ethnicity, 169 (77.6%) patients were Caucasians, 45 (20.6%) were mestizos, and 4 (1.8%) patients were Black. In local general population, according to Brazilian Institute of Geography and Statistics–IBGE 2010 Census[13], above 20 years of age, there are 742,139 Caucasians (42.63%), 144,901 Blacks (8.32%) and 818,212 Mestizos (47%).

BMIs at disease onset were calculated for 138 patients from their medical records and drug-dispensing records and ranged between 14.9 and 34.9 kg/m^2^, with an average of 23.5±4.1 kg/m^2^, without significant difference between the sexes (p = 0.15).

The IDHM-2010 ranged between 0.577 and 0.955, with an average of 0.83, without significant difference between the sexes (p = 0.46).

Family history of the disease was reported by 11 (5%) patients.

The mean periods of diagnosis from symptom onset were 22.7 months in men and 23.5 months in women.

In the same period, SIM/DATASUS data indicated the occurrence of 162 deaths due to ALS in the region. Of these deaths, 99 cases were also present in the CRDN database. The deaths occurred with similar frequencies between 2005 and 2014, except in 2007, when nine deaths were reported. The highest frequency of deaths occurred in the age groups 60–69 years and 70–79 years, accounting for 59.8% of deaths. No deaths were reported in the age group <30 years. Deaths occurred in 90(55.6%) men and 72 (44.4%) women. The mean survival periods were 45.7±47.0 months among men and 39.3±29.8 months among women, without significant difference between the sexes (p = 0.26).

Table 1 presents the distribution of patients according to the form of presentation. The most frequent form of presentation involved signs and symptoms in the upper limbs, followed by symptoms in the lower limbs and in bulbar muscles. The average age at disease onset did not differ significantly among the spinal or bulbar-onset forms (p = 0.06).

**Table 1:** Amyotrophic Lateral Sclerosis patients profile considering the site of onset, age and gender.

Site of Onset	Age average (SD)^*^	CI 95%	Men (%)	Women (%)	Total (%)
** Arm**	55.1 (12.9)	52.5–57.7	62 (47)	36 (41.9)	98 (45)
**Leg**	58.2 (12.0)	55.5–61.0	48 (36.4)	29 (33.7)	77 (35.3)
**Bulbar**	60.0 (10.6)	56.7–63.3	22 (16.7)	21 (24.4)	43 (19.7)
**Total**	57.2 (12.3)	55.6–58.86	132 (100)	86 (100)	218

The frequency of disease presentation was similar between the sexes. The frequency of impairment of another body region is shown in Table 2. Impairment of the contralateral limb was more frequent, followed by impairment of the ipsilateral limb and bulbar region. These frequencies were not different between the sexes.

**Table 2:** Clinical progression in patients with Amyotrophic Lateral Sclerosis

Clinical Progression	Male(%)	Female(%)	Total
**Contralateral**	87(65.9)	48(55.8)	135(61.9)
**Ipsilateral**	40(30.3)	35(40.7)	75(34.4)
**Bulbar**	5(3.8)	3(3.5)	8(3.7)
**Total**	132	86	218

Electroneuromyography (ENMG) was performed on all patients, and the diagnosis was confirmed in 181 cases (83%). However, in 18(8.3%) cases initially classified as Suspected, the procedure did not confirm the diagnosis in 55.6% cases. Moreover, ENMG did not confirm the diagnosis of the bulbar-onset form in 12 (27.9%) patients, of which 15.3% cases involved the upper limbs and 13% cases involved the lower limbs.

The following signs were observed in the course of the disease: pyramidal in 153(70.2%) patients; dementia in 16(7.3%) patients; Urinary disorders in 5(2,3%) of which 2 (0.9%) and 3(1.4%) presented with incontinence and urinary retention, respectively, not explained by urologic disease.

The analysis of the unadjusted risk ratio shown in Table 3 indicates that the following variables were not statistically significant and were excluded: sex, IDHM-2010, clinical progression, presence of pyramidal signs, family history, urinary disorders, dementia, smoking, alcoholism, exposure to pesticides and heavy metals, intense physical activity, and diagnostic delay (S1 Table).

**Table 3:** Crude and adjusted Risk Ratio for survival, by demographic and selected clinical variables and adjusted^a^ by age, BMI and site of onset.

	Risk Ratio—RR (CI 95%)			
	Crude	p-value	Adjusted^a^	p-value
**Age (years)**		0.01		0.001
** **< 50	1	-	1	-
** **51–60	1.53 (0.66–3.56)	0.31	1.37 (0.59–3.19)	0.46
** **61–75	1.83 (0.80–4.17)	0.15	1.90 (0.83–4.39)	0.13
** **> 75	6.70 (2.02–22.24)	0.001	12.47 (3.51–44.26)	< 0.0001
**BMI (kg/m** ^**2**^ **)**		0.03		0.006
** **< 25	2.59 (1.08–6.23)	0.03	3.56 (1.44–8.86)	0006
** **≥ 25	1	-	1	-
** Site of onset**		0.003		0.0002
** **Spinal	1	-	1	-
** **Bulbar	2.97 (1.44–6.14)	0.003	4.56 (2.06–10.12)	0.0002

The Cox multivariate regression model indicated that only age, BMI, and the initial form of presentation were risk factors significantly associated with the survival period (Table 3). The risk of death among patients aged >75 years was approximately 12-fold higher (relative risk [RR]: 12.47, p<0.0001) than that of patients aged <50 years. In contrast, the risk of death among patients in the age groups 51–60 and 61–75 years did not differ significantly compared with the age group <50 years. The risk of death among patients with a BMI <25 kg/m^2^ was approximately 3.5-fold higher than that of patients with a BMI ≥25 kg/m^2^ (RR: 3.56, p = 0.006). The risk of death among patients with bulbar-onset ALS was approximately 4.5-fold higher (RR: 4.56, p = 0.0007) than that of patients with spinal-onset ALS. No differences were observed in relation to the onset sites in the upper and lower limbs.

**Fig 2:**
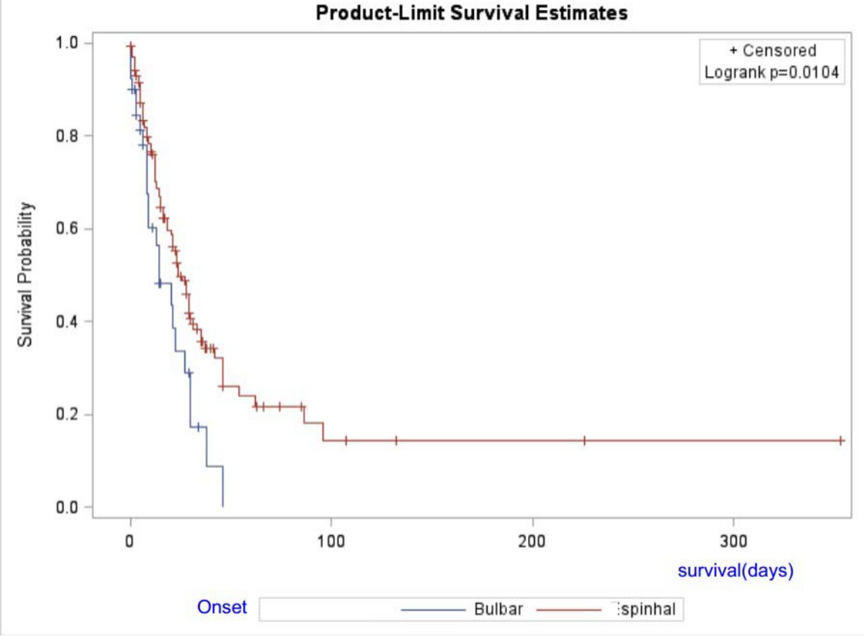
Survival of Amyotrophic Lateral Sclerosis patients taking riluzole in Federal District.

The results of the log-rank test, shown in Fig 2, indicate that the survival period in patients with spinal-onset ALS was significantly longer than that in patients with bulbar-onset ALS (p = 0.01). Accordingly, approximately 70% of the patients with spinal-onset ALS were still alive one year after initiation of follow-up, whereas only 56% of those with bulbar-onset ALS were still alive in the same period.

## Discussion

The observation of clinical events is important for the evaluation of prognosis in ALS, which has been previously demonstrated [14–16]. Most studies have reported that age, bulbar-onset ALS, and cervical weakness are factors associated with poor prognosis and lower survival, whereas longer diagnostic delay and use of riluzole were positively correlated with increased survival [14–17].

The study was conducted in a population ethnically distinct from populations living in Europe and North America [13]. We noted that the average age of 57.2±12.3 years at disease onset was similar in both sexes and was below the average age of populations from the northern hemisphere (61.8±3.8 years)[1]. In addition, 50% of the study patients were aged between 48 and 66 years. This finding was corroborated by other studies in Latin America [18–20] and may indicate the presence of yet unidentified genetic mutations or environmental factors involved in the disease etiology.

Both, genetic and environmental factor are still unclear in ALS. It was related [21] a repeat expansion in C9orf72 hexanucleotide in 6% of sporadic ALS and 44% of familiar ALS patients of European ancestry, with younger age at onset, shorter disease duration and higher frequency of Frontotemporal Dementia, in different ethnic populations. With respect to environmental factors, to our knowledge, in Latin America has no study.

With regard to ethnicity, the disease predominated in Caucasians (77.6%); however, the differences in the survival rate were not significant between the ethnicities. Similar findings have been previously reported [22–24]. In contrast, an Asian study monitored 73 patients over a 10-year period and reported a lower survival rate among Ethnic Indians compared with other ethnic groups [24].

It was observed that the risk of death among patients aged >75 years was approximately 12-fold higher (RR: 12.47, p<0.0001) than that of patients aged <50 years. In contrast, lower survival was not observed in the age group 51–75 years compared with the age group <50 years. A recent study in the United States evaluated 456 incident cases and reported that the survival rate decreased linearly with the increase in age [25].

This same study [25] reported survival rates of 67% at 12 months and 46% at 24 months. In the same period, it was observed survival rates of 70% for spinal-onset ALS and 56% for bulbar-onset ALS. Previous studies have reported better prognosis when the onset site was in the lower limbs [26, 27] or bi brachial variant [28]. However, the present study found that the risk of death in bulbar-onset ALS was 4.5-fold higher (RR: 4.56, p = 0.0007) than that of spinal-onset ALS, independently of whether the onset site was in the arms or legs.

A Scottish study involving 1,226 patients [3] also reported worse prognosis associated with bulbar-onset ALS—although of lower magnitude (hazard ratio [HR]: 1.25, 95% confidence interval [CI]: 1.09–1.46)—presence of pyramidal signs (HR: 1.23, 95% CI: 1.01–1.49), and advanced age. The present study did not observe worse prognosis with the presence of signs of impairment of the first motor neuron.

There is controversy with regard to the association between dementia and ALS prognosis. In 2011, a study evaluated the association between motor neuron disease and frontotemporal dementia and indicated that the presence of signs of dementia in the frontal lobe were associated with bulbar-onset ALS and lower survival [29], however, this result was not observed in other studies [30]. In this series, it was observed related dementia in 7.3% cases; however, there was no increased risk of death in these patients.

A study conducted in Taiwan reported a correlation between low socioeconomic status and lower survival rates [31]. In the Federal District, the socioeconomic factors included in the Brazilian IDHM corresponded to the same three variables contemplated in the global HDI: longevity, education, and income. No significant difference in survival was observed between the regions with higher and lower IDHN, but this difference was higher than 0.8, a value considered high for Brazil.

No correlation was observed between survival period and longer diagnostic delay; however, in our study, the diagnostic delay was longer (averages of 22.74 months among men and 23.50 months among women) compared with other studies, wherein the average delay was <15 months [6,32,33].This result may have occurred because the patients were sent to the CRDN only after confirmation of the diagnosis by ENMG.

Nerve conduction and electromyography studies (ENMG) are important tools for the identification of the degree and extent of impairment of the lower motor neurons and neuronal loss [34, 35] and to discard diagnoses of other diseases; however, in the present study, the examination failed to confirm the disease in 55.6% of cases initially classified as suspicious and in 27.9% of cases of bulbar-onset ALS, which reinforces the assumption that the clinical events should always be considered. A recent study in France [34] showed that the maximum sensitivity of the ENMG for bulbar-onset ALS was 49.98% using the criteria of Awaji-shima.

No significant difference in survival was observed between the sites of disease progression in the ipsilateral limbs, contralateral limbs, or bulbar region. A Japanese study involving 150 patients reported that the average survival rate was lower when bulbar manifestations were present in the first year of disease onset (p<0.001), in cases of rapid progression to another onset site, and when symptoms progressed with longitudinal or ipsilateral patterns [27]. The results of Turner [24] reinforce the hypothesis that the period of progression to another site is more important for disease prognosis than the anatomical distribution.

The poorer prognosis in the disease forms that prematurely affect the bulbar muscles may be due to patient malnutrition. In the present study, it was found that the survival rate of patients with a BMI <25 kg/m^2^ was approximately 4-fold lower (RR = 3.56, 95% CI: 1.44–8.86) than that of patients with a BMI ≥25 kg/m^2^. Recent studies have indicated that poor nutritional status at the beginning and in the course of disease was associated with worse prognosis. A French study involving 63 patients indicated that the mean disease duration was lower among those with weight loss >10% during the disease course [35]. Other studies have shown that a BMI of 30–35 kg/m^2^ was associated with a higher survival rate and that the rapid decrease in this index was associated with poor prognosis. Although these studies suggest that weight gain may have a protective effect in ALS, they did not confirm that an excessively high BMI was beneficial [36–39].

With regard to environmental factors, no correlations were detected between alcohol consumption, smoking, contamination with pesticides and heavy metals, intense and prolonged exercise, malignancy, and disease prognosis. In a population-based study conducted in the Netherlands with 494 patients with ALS and 1,599 controls [40], a multivariate analysis indicated increased risk for ALS and lower survival rates among smokers (HR: 1.51, 95% CI: 1.07–2.15). These factors were evaluated because of the possible correlation between the occurrence of oxidative stress and disease pathogenesis in which DNA injury mechanisms and pathological changes in lipid and protein metabolism may lead to cell death via necrosis or apoptosis. However, the roles of these factors on disease prognosis have not been characterized when a degenerative process has already been established [40, 41].

It was observed that 5% of patients had a family history for the disease, a frequency that is in accordance with the incidence of the familial forms of the disease in most studies, with a range between 1.6% and 5.6% [32], but this variable did not influence disease prognosis. Finding a precise genetic diagnosis in ALS is difficult because of the large phenotypic variability of the disease. Several studies have described mutations in more than 22 genes; in addition, more than one mutation may be present in the same patient, and most of these mutations may have high or moderate penetrance. The most frequent mutations reported encode the enzyme copper/zinc superoxide dismutase (SOD1), transactive-response DNA-binding protein 43 (TARDBP), C9orf72In, ubiquitin-like protein 2 (UBQLN2), profilin 1 (PFN1), optineurin (OPTN), angiogenin (ANG), and the RNA-binding protein FUS (fused in sarcoma)[42,43].

In conclusion, the factors that most impacted prognosis and decreased the survival rate in ALS were age >75 years, BMI <25 kg/m^2^, and bulbar-onset ALS. For the earliest possible diagnosis and interventions aimed at prolonging survival, the consideration of clinical events rather than electromyography is essential, primarily in cases of bulbar-onset ALS and in cases classified as suspect according to the El Escorial diagnostic criteria.

## Supporting Information

S1 Table SurvivalofALS: Survival of patients with ALS in the Federal District with respect to clinical and demographic variables (DOCX)
