# Supplementary material for: Prognostic Factors in Amyotrophic Lateral Sclerosis: A Population-Based Study
Source: PLoS One. 2015 Oct 30;10(10):e0141500. doi: 10.1371/journal.pone.0141500 (PMC4627754; doi:10.1371/journal.pone.0141500)
Supplement: S1 Table — (DOCX) [file pone.0141500.s001.docx]

|  | **Risk Ratio - RR (CI 95 %)** | | | |
| --- | --- | --- | --- | --- |
|  | **Crude** | **p-value** | **Adjusted^a^** | **p-value** |
| **Age(years)** |  | 0.0199 |  | 0.0013 |
| < 50 | 1 | - | 1 | - |
| 51 - 60 | 1.53 (0.66 – 3.56) | 0.3194 | 1.37 (0.59 – 3.19) | 0.4602 |
| 61 – 75 | 1.83 (0.80 – 4.17) | 0.1515 | 1.90 (0.83 – 4.39) | 0.1306 |
| > 75 | 6.70 (2.02 – 22.24) | 0.0019 | 12.47 (3.51 – 44.26) | < 0.0001 |
| **BMI (Kg/m^2^)** |  | 0.0331 |  | 0.0062 |
| < 25 | 2.59 (1.08 – 6.23) | 0.0331 | 3.56 (1.44 – 8.86) | 0.0062 |
| ≥ 25 | 1 | - | 1 | - |
| **Site of Onset** |  | 0.0032 |  | 0.0002 |
| spinal | 1 | - | 1 | - |
| Bulbar | 2.97 (1.44 – 6,14) | 0.0032 | 4.56 (2.06 – 10.12) | 0.0002 |
| **Gender** |  | 0.6795 |  |  |
| Female | 1 |  |  |  |
| Male | 1.15 (0.60 – 2.18) | 0.6795 |  |  |
| **IDH** |  | 0.7220 |  |  |
| 0 – 0,669 | 1 | - |  |  |
| 0,670 – 0,735 | 0.65 (0.17 – 2.43) | 0.5223 |  |  |
| 0,736 – 0,786 | 0.89 (0.31 – 2.55) | 0.8242 |  |  |
| 0,787 – 0,853 | 1.12 (0.39 – 3.18) | 0.8349 |  |  |
| 0,854 – 1,000 | 1.11 (0.40 – 3.10) | 0.8437 |  |  |
| **Clinical progression** |  | 0,3942 |  |  |
| Crossed and Ipsilateral | 1 | - |  |  |
| Bulbar | 1.89 (0.44 – 8.22) | 0.3942 |  |  |
| **Pyramidal Syndrome** |  | 0.8867 |  |  |
| No | 1 | - |  |  |
| Yes | 1.39 (0.70 – 2.77) | 0.8867 |  |  |
| **Confirmatory EMG*** |  | 0.9442 |  |  |
| No | 1 | - |  |  |
| Yes | 1.02 (0.57 – 1.82) | 0.8867 |  |  |
| **Familial** |  | 0.4218 |  |  |
| Yes | 1 | - |  |  |
| No | 2.26 (0.31 – 16.49) | 0.4218 |  |  |
| **Urinary Symptoms** |  | 0.9914 |  |  |
| No | 1 | - |  |  |
| Yes | 0.00 | 0.9914 |  |  |
| **Dementia** |  | 0,7183 |  |  |
| Yes | 1 | - |  |  |
| No | 1.14 (0.55 – 2.35) | 0.7183 |  |  |
| **Alcohol consumption** |  | 0.9346 |  |  |
| Yes | 1 | - |  |  |
| No | 1.02 (0.57 – 1.83) |  |  |  |
| **Tabagism** |  | 0.7751 |  |  |
| No | 1 | - |  |  |
| Yes | 1.08 (0.65 – 1.78) | 0.7751 |  |  |
| **Pesticides** |  | 0.8107 |  |  |
| Yes | 1 | - |  |  |
| No | 1.09 (0.55 – 2.14) | 0.8107 |  |  |
| **Exposure to Metals** |  | 0.9757 |  |  |
| Yes | 1 | - |  |  |
| No | 1.02 (0,31 – 3,33) | 0.9757 |  |  |
| **Atlhete** |  | 0.9880 |  |  |
| No | 1 | - |  |  |
| Yes | 1289410 (0,00 -) | 0.9880 |  |  |
| **Time to diagnosis delay (months)** |  | 0.3836 |  |  |
| **≤ 12** | 1.78 (0.77 – 4.02) | 0.1817 |  |  |
| **13 – 24** | 1.26 (0.54 – 2.93) | 0.5942 |  |  |
| **> 24** | 1 | - |  |  |

*Electromyography

S1 Table- Crude and Adjusted Risk Ratio (RR) analyzing Survival of patients with ALS in the Federal District with respect to clinical and demographic variables.
